# Post-hypnotic safety suggestion improves stress coping with long-lasting effects

**DOI:** 10.1038/s41598-024-54071-3

**Published:** 2024-02-12

**Authors:** Barbara Schmidt, Nicolas Rohleder, Veronika Engert

**Affiliations:** 1 Institute of Psychosocial Medicine, Psychotherapy and Psychooncology, Jena University Hospital, Stoystraße 3, 07743 Jena, Germany; 2 Chair of Health Psychology, Institute of Psychology, Friedrich-Alexander-Universität, Erlangen-Nürnberg, Germany; 3 Max Planck Institute for Human Cognitive and Brain Sciences, Leipzig, Germany

**Keywords:** Hypnosis, Suggestion, Stress, Anxiety, Coping, Long-term effect, Emotion, Stress and resilience, Diagnostic markers, Predictive markers, Endocrinology

## Abstract

Effective coping with acute stress is important to promote mental health and to build stress resilience. Interventions improving stress coping usually require long training periods. In this study, we present a hypnosis-based intervention that produces long-term effects after a single hypnosis session. In that session, we established a post-hypnotic safety suggestion that participants can activate afterwards with a cue, the Jena Safety Anchor. We tested 60 participants in our study who all received the hypnosis session and a stress task. The safety group used the Jena Safety Anchor during acute stress (Trier Social Stress Test, TSST). The control group used a neutral anchor. We measured subjective stress responses via self-reports and physiological stress responses via saliva and blood samples as well as heart rate. One week later, all participants filled in an online survey to measure long-term effects of the post-hypnotic safety suggestion. We found that participants using the Jena Safety Anchor during the TSST reported significantly lower stress compared to the control group. The safety group also reported significantly fewer negative thoughts concerning their TSST performance than the control group during the stress recovery phase and 1 week later. All participants indicated that the Jena Safety Anchor still worked 1 week after its establishment. Suggestibility did not affect the efficacy of the Jena Safety Anchor. Our findings demonstrate that post-hypnotic safety suggestions improve stress coping with long-lasting effects, which makes it a promising intervention to promote mental health and establish stress resilience in just one hypnosis session.

## Introduction

Chronic stress is a leading risk factor for diseases and can lead to affective and cardiovascular disorders, among others^1^. Being able to cope with acute stress is an important skill promoting mental health^2^. The general ability to bounce back from stress is termed resilience. Next to lower stress levels, resilience is associated with further positive outcomes like less negative affect, more positive affect, and fewer physical symptoms^3^. To address the increasing levels of stress and stress-related disease in our society^4^, there is an urgent need to develop effective interventions that help us cope with stress. One option is mindfulness-based meditation, but it takes long training periods and high personal investment until it effectively improves stress coping^5–8^. A recent study used a combined mindfulness/hypnosis audio intervention that participants used daily for 1 week and found that it reduced stress reactivity compared to an active control group^9^. An intervention that can reduce stress-related symptoms with less commitment would therefore be desirable. Recent results show that one hypnosis session can alleviate mental stress in chronically stressed individuals^10,11^, but it is unclear whether it can likewise help participants deal with acutely stressful situations.

Hypnosis is a specific state of consciousness characterized by focused attention that increases our capacity to respond to suggestions^12^. A hypnotic state is usually started and ended by a hypnosis therapist. Most hypnosis paradigms use specific suggestions to modify perception and behavior. When suggestions are both given and tested during hypnosis, they are referred to as hypnotic suggestions. Numerous studies have shown that a specific suggestion is associated with suggestion-specific neuronal, behavioral and psychological effects. For example, the suggestion to have a wooden board in front of your eyes leads to lower ERP amplitudes to visual stimuli^13,14^, and the suggestion to wear earplugs leads to lower ERP amplitudes to sounds^15^.

To induce a feeling of safety during hypnosis, the hypnotist guides participants to imagine being at a place where they feel safe, such as lying at a sunny beach or sitting on their mother’s lap as a child. No matter what place participants choose, they are guided to imagine every sensory detail of the situation like the specific smell, the feeling of the sun on their skin, a warm summer breeze etc. Studies show that when participants feel safe during hypnosis, ERP amplitudes to monetary rewards are reduced^16,17^. In the medical context, hypnotic suggestions of safety reduce opioid use^18^ and help patients to better accept challenging medical procedures like non-invasive ventilation^19–21^. Thus, the suggestion of being in a safe place is a promising method to also reduce acute stress sensitivity.

In studies using hypnotic suggestions, the state of hypnosis and the effect of the specific suggestions occur at the same time. That makes it difficult to disentangle the relaxing effect of the hypnotic state (“I am in hypnosis”) and the effect of the specific suggestion (“I feel safe”). As a solution to this problem, it is possible to associate the effect of a specific suggestion with an anchor to elicit the suggestion effect outside of the hypnotic state. This method is called post-hypnotic suggestion. A major advantage of post-hypnotic suggestions is their longevity. Once established, the effect can last for weeks, as we have shown in our laboratory^22^. As eliciting anchor, participants wrote the letter S—for safety—on a piece of paper while in a hypnotic state. To re-activate the feeling of safety after hypnosis, they simply looked at the paper, folded it and put it in their pocket. The hypnotist provided no further information. Only the S paper elicited the feeling of safety, and this effect lasted for weeks after the initial hypnosis session^22^. We call this post-hypnotic anchor Jena Safety Anchor.

In the present study, we use the Jena Safety Anchor in a situation of acute stress. All participants received hypnosis with safety suggestion and established the Jena Safety Anchor. Half of the participants were assigned to the safety group and used the S paper in the acute stress task. The other half of participants did not use the Jena Safety Anchor. They had to hand it to the experimenter and received a neutral paper instead. Our main hypothesis was that stress will be reduced in the safety group that used their Jena Safety Anchor during acute stress compared to the control group.

## Materials and methods

**Figure 1:**
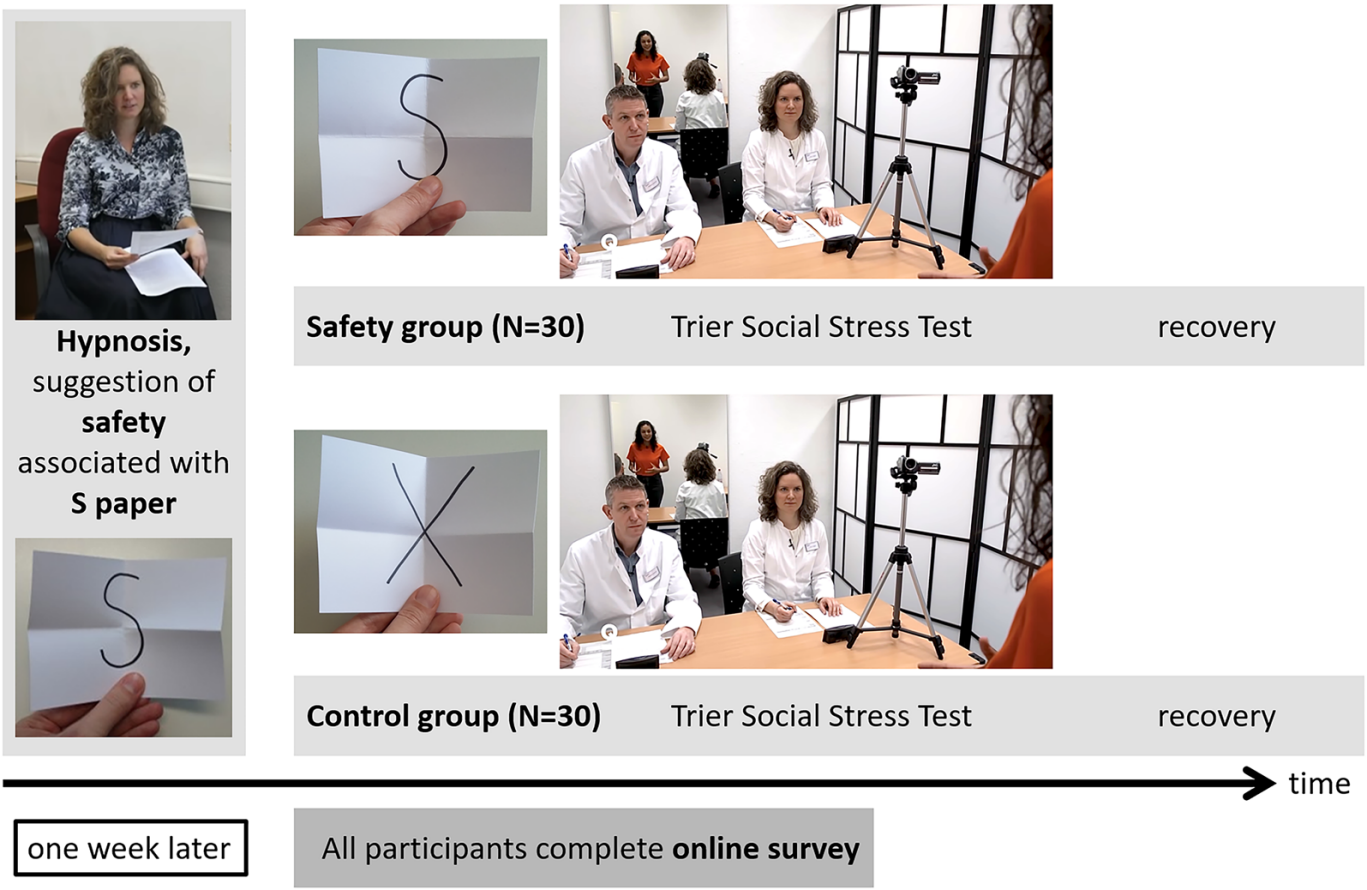
Experimental design of the study. Participants in the safety group used the post-hypnotic safety anchor (Jena Safety Anchor, S paper with the letter S for safety on it) during acute stress; participants in the control group used a neutral anchor (X paper). All participants went home after the main experimental session with their S paper. One week later, all participants filled in an online survey to measure long-time effects of the S paper. The first author of the manuscript was the hypnotist, but never part of the TSST jury.

In the present study, we first established the post-hypnotic safety anchor (Jena Safety Anchor) in a hypnosis session and then induced acute stress via a standardized stress paradigm, the Trier Social Stress Test (TSST^23^). As a neutral control anchor, all 60 participants wrote the letter X on a piece of paper (X paper) at the beginning of the main experimental session. After establishing the Jena Safety Anchor (S paper) during hypnosis, all participants handed their S paper to the hypnotist. Just before the acute stress task (TSST) started, participants in the safety group (N = 30) were given back their safety-anchored S paper. Participants in the control group (N = 30) received their neutral X paper. All participants looked at their paper, folded it and put it in their pocket. The allocation of participants to the experimental groups was randomized. After the acute stress task (TSST), we traced participants’ stress recovery for 90 min. At the end of the main experimental session, all participants went home with their safety-inducing S paper, no matter which experimental group they belonged to. One week later, we re-contacted all participants and asked for their ongoing feelings of safety elicited by the Jena Safety Anchor, as well as for ongoing positive and negative thoughts in association with the experienced stress situation. Figure 1 shows the experimental design of the study.

To measure participants’ stress responses during the main experimental session, we analyzed both, subjective markers, such as perceived stress and anxiety, and physiological markers, such as adrenaline, cortisol and heart rate. Please note that we also assessed other markers in participants’ blood plasma samples that can be seen in our Zenodo repository (10.5281/zenodo.10561059). These are: Noradrenaline, Dopamine, CRP, IL-1, IL-6. These markers were measured exploratory and are not part of the current analysis and manuscript. Generally, we expected low stress levels after hypnosis, increased stress levels during acute stress and normalization of stress levels in the 90-min post-stress recovery period. Concerning the effect of the Jena Safety Anchor (S paper), we expected lower stress and anxiety ratings as well as lower adrenaline, cortisol and heart rate increases during acute stress and in the recovery period in the safety compared to the control group. In line with Böhmer and Schmidt^22^, we expected that the Jena Safety Anchor would still elicit the feeling of safety 1 week after its establishment.

### Participants

We conducted a power analysis with G*power to determine the adequate sample size for our study. We based this power analysis on our main hypothesis, which is the group difference in stress responses, measured immediately after the TSST. The effect size of previous hypnotherapeutic interventions on subjective evaluations conducted in our laboratory were at least *d* = 0.7. With a power level of 0.85 and an alpha level of 0.05, 60 participants (30 participants in each group) are required in a between-group design according to G*power^24^. For anxiety ratings, we computed an additional power analysis that focused on the interaction effect of group (safety, control) and measurement time (before TSST, after TSST). To measure an interaction effect of η_p_^2^ = 0.14 with a power level of 0.85 and an alpha level of 0.05, we calculated a necessary sample size of 58 participants. Participants were recruited via mailing lists, postings on social media and in lectures. Thus, we obtained data of 60 participants (30 female, 30 male) that were pre-tested concerning their suggestibility in a live session with the Harvard Group Scale of Hypnotic Susceptibility (HGSHS^25^), conducted by the first author of the study. We invited 20 participants with a HGSHS score of 8–12 (high hypnotic suggestibility), 20 participants with a HGSHS score of 5–7 (middle hypnotic suggestibility), and 20 participants with a HGSHS score of 0–4 (low hypnotic suggestibility). Each experimental group (safety and control) consisted of five female participants with high, middle and low suggestibility and five male participants with high, middle and low suggestibility. The mean age of participants was 25.9 years (SD = 8.0 years, range 18–59 years) and they were predominantly white Europeans. For the main experiment, female participants had to be in the luteal phase of their natural menstrual cycle, as recommended for stress studies including cortisol measurements^26^. General inclusion criteria were that participants are at least 18 years old, do not smoke, do not have acute psychological or neurological problems and do not suffer from cardiovascular disease. The main experimental session always started at 2 p.m. including hypnosis, the post-hypnotic suggestion and the TSST. Before the main session, we instructed participants not to drink alcohol and eat spicy food starting the day before, to drink only still water and not exercise one hour before the session starts. In the beginning of the main experimental session, participants consumed a small snack (apple juice or chocolate bar). The main session lasted about 3 h; the second session was an online survey taking about 30 min 1 week after the first session. Participants received 20 Euros for participation. The local ethics committee at the Jena University Hospital approved the study (2021-2253-BO). All methods were performed in accordance with the relevant guidelines and regulations.

### Procedure

After we screened participants with the HGSHS^25^ to assess their suggestibility, they filled in two online questionnaires to measure their trait anxiety (State Trait Anxiety Inventory, STAI-T^27^, possible values between 20 and 80) and chronic stress (Short Screening Scale for Chronic Stress (SSCS)^28^, possible values between 0 and 48). Trait anxiety and chronic stress did not differ between experimental groups (p > 0.1) and the average scores per group reflected medium levels of trait anxiety (mean STAI-T score: 41.7, SD: 9.9) and medium levels of chronic stress (mean SSCS score: 21.1, SD: 8.6) in our sample.

For the main experimental session, participants arrived at the laboratory at 2 pm. They read the study information sheet and signed the informed consent statement. To create a neutral anchor, all participants wrote the letter X on a piece of paper. Then, we offered them to drink a small bottle of apple juice or eat a chocolate bar to increase and normalize their blood sugar levels. We helped participants to put on a Polar H10 chest belt to measure their heart rate. A trained medical student then placed a permanent venous catheter in the non-dominant arm of participants and infused saline solution.

### Hypnosis session

The participants went to a separate testing room after the preparations with the first author of the study. Both sat down in a comfortable chair and the first author of the study started the hypnosis session with a hypnosis introduction following the Stanford Hypnotic Susceptibility Scale (SHSS^29^). To test if participants followed her hypnotic suggestions, she used the first item of the SHSS, suggesting that there is a heavy weight in the participants’ right hand. When the right hand is sinking downwards, participants passed the test. Then, the hypnotic suggestion of safety started. The hypnotist guided the participants to imagine that they are at a place where they feel comfortable and safe. Then she instructed participants to open their eyes and write the letter S for safety on a piece of paper in front of them. The hypnotist suggested that every time they see this paper with the letter S on it, fold it and put it in their pocket, they would feel safe again. Then, the hypnotist ended the hypnotic state. The duration of the hypnosis intervention was about 30 min. Please see our Zenodo repository (10.5281/zenodo.10561059) for the complete wording of the hypnosis intervention. All participants were hypnotized by the first author of the study which was the only hypnotherapist. After that, participants indicated how safe they feel on a scale from 1 (no change) to 5 (very safe). They also filled in the Inventory Scale of Hypnotic Depth (ISHD^30^) containing 36 items to measure their trance depth. Participants rated each item on a scale from 1 to 4, resulting in a maximal score of 144. Participants now handed their S paper to the first author of the study. In the following, participants indicated how anxious they are with the STAI-S^27^ (possible values between 20 and 80) and how stressed they feel on a visual analogue scale (VAS) from 0–100. Then, the trained medical student took the first blood and saliva samples using monovettes and salivettes (Sarstedt).

### Safety and control group split

Group randomization was performed by an odd/even allocation based on participants’ id, stratified by the suggestibility and sex of participants to ensure the same amount of male/female and low/middle/high suggestible participants in each group. The first author of the study gave participants of the safety group their Jena Safety Anchor back that they established during the hypnosis session, their S paper. Participants in the control group received the neutral anchor that they had written in the beginning of the session, the X paper. All participants looked at the letter on the piece of paper, folded it and put it in their pocket.

### Acute stress induced with Trier Social Stress Test (TSST)

An experimenter introduced the participants to the mock expert jury and told the participants that they should now prepare for a job interview for five minutes, sitting at a separate table. The male and female TSST juror already sat behind a desk in white coats and observed the participants during the TSST preparation period. After five minutes, the jury members told the participants that their preparation time was over and instructed them to stand in a marked square, adjusted the camera to film the presentation and asked the participants to start their free speech. Participants could see themselves in a mirror placed behind the two jurors. The jury gave no feedback and responded minimally while interacting with the participants using standardized sentences. After five minutes, the jury told the participants that they should now switch to the math test, subtracting in steps of 17 from the number 2023. Every time the participants made a mistake, they had to start with the number 2023 again. After five minutes, the jury told the participants that the test was over, stopped the camera recording and left the room.

### Recovery period

Immediately after the TSST, the medical student took the second blood and saliva sample and asked the participants to fill in the state anxiety questionnaire (STAI-S) and the VAS stress scale. Participants then indicated how safe they felt on a scale from 1 (no change) to 5 (very safe).

The medical student took further saliva samples at 10 min, 20 min, 30 min and 45 min after the TSST. She also took two more blood samples 20 min and 90 min after the TSST. Every time she took a sample, participants also filled in the VAS stress scale from 1–100. Half an hour after the TSST, participants indicated their positive and negative thoughts regarding their performance in the TSST using the thoughts questionnaire^31^.

### Second experimental session (1 week later)

One week after the main experimental session, participants completed an online questionnaire. Participants again indicated how safe they feel with their Jena Safety Anchor on a scale from 1 (no change) to 5 (very safe). Then, they completed the thoughts questionnaire to measure their positive and negative thoughts concerning their TSST performance the week before.

## Results

**Figure 2:**
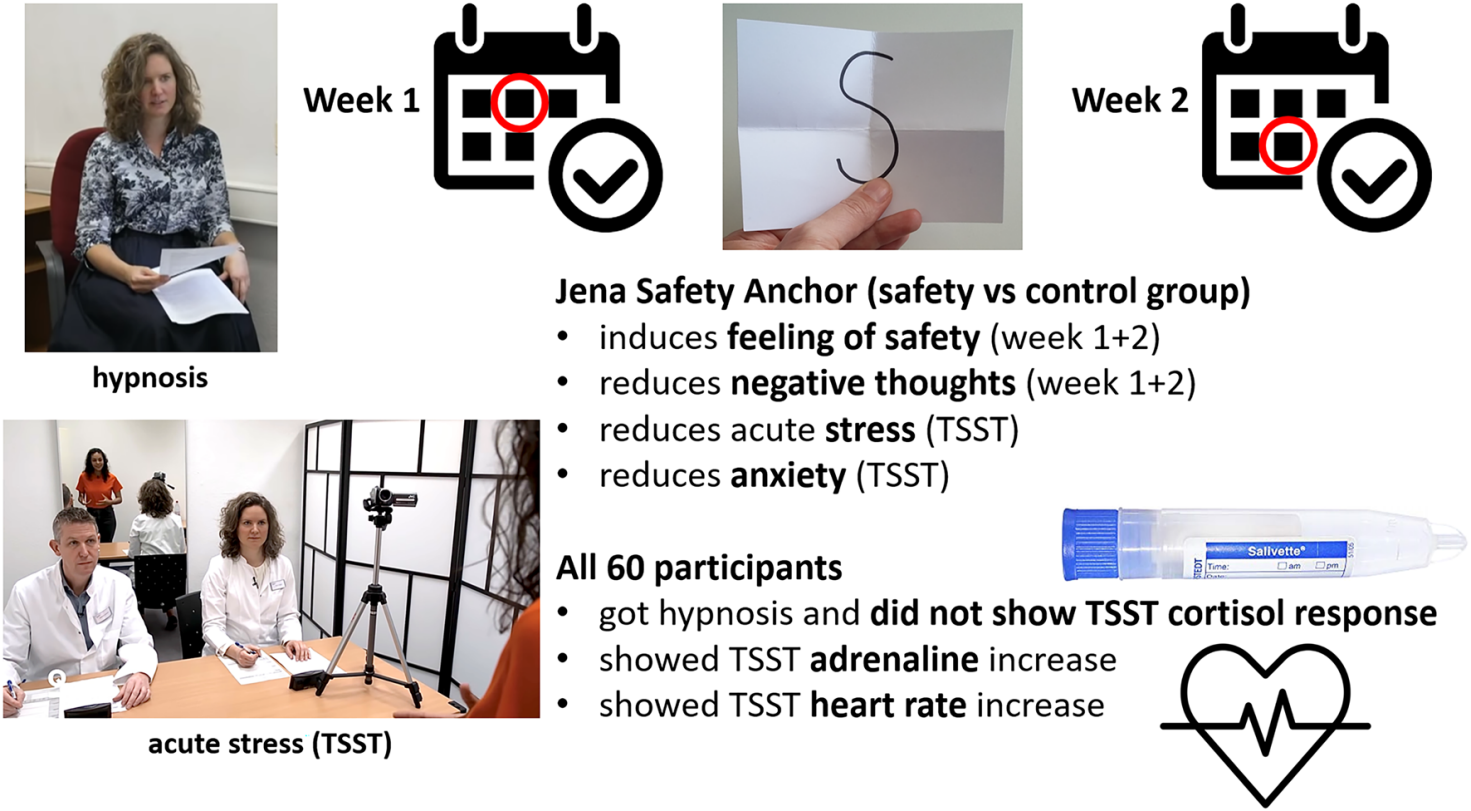
Main results of the study. Participants in the safety group felt less stressed and anxious after acute stress and during recovery compared to the control group. Adrenaline levels and heart rate increased during acute stress and normalized afterwards without significant group differences. Cortisol levels did not rise over 1.5 nmol/l on average after acute stress in all participants. The effect of the post-hypnotic safety suggestion was stable over 1 week irrespective of experimental group. The first author of the manuscript was the hypnotist, but never part of the TSST jury as depicted in the picture.

Our data show that the post-hypnotic suggestion of safety (S paper) led to a significant reduction in stress ratings immediately after acute stress and during the recovery period. In addition, participants of the safety group had fewer negative thoughts concerning their performance in the TSST. Adrenaline levels peaked immediately after acute stress but did not differ significantly between groups. Likewise, heart rate increased during acute stress and normalized during stress recovery but showed no significant group difference. Cortisol levels did not rise higher than 1.5 nmol/l on average in either of the groups, which is an established criterion for physiologically significant cortisol release^32^. One week after the stress test, all participants indicated that the S paper still elicited a feeling of safety. Also, participants of the safety group still had significantly fewer negative thoughts concerning their performance in the TSST compared to the control group. Figure 2 gives an overview over the main predicted and observed effects of the study.

### Lower stress and anxiety ratings with post-hypnotic safety suggestion

**Figure 3:**
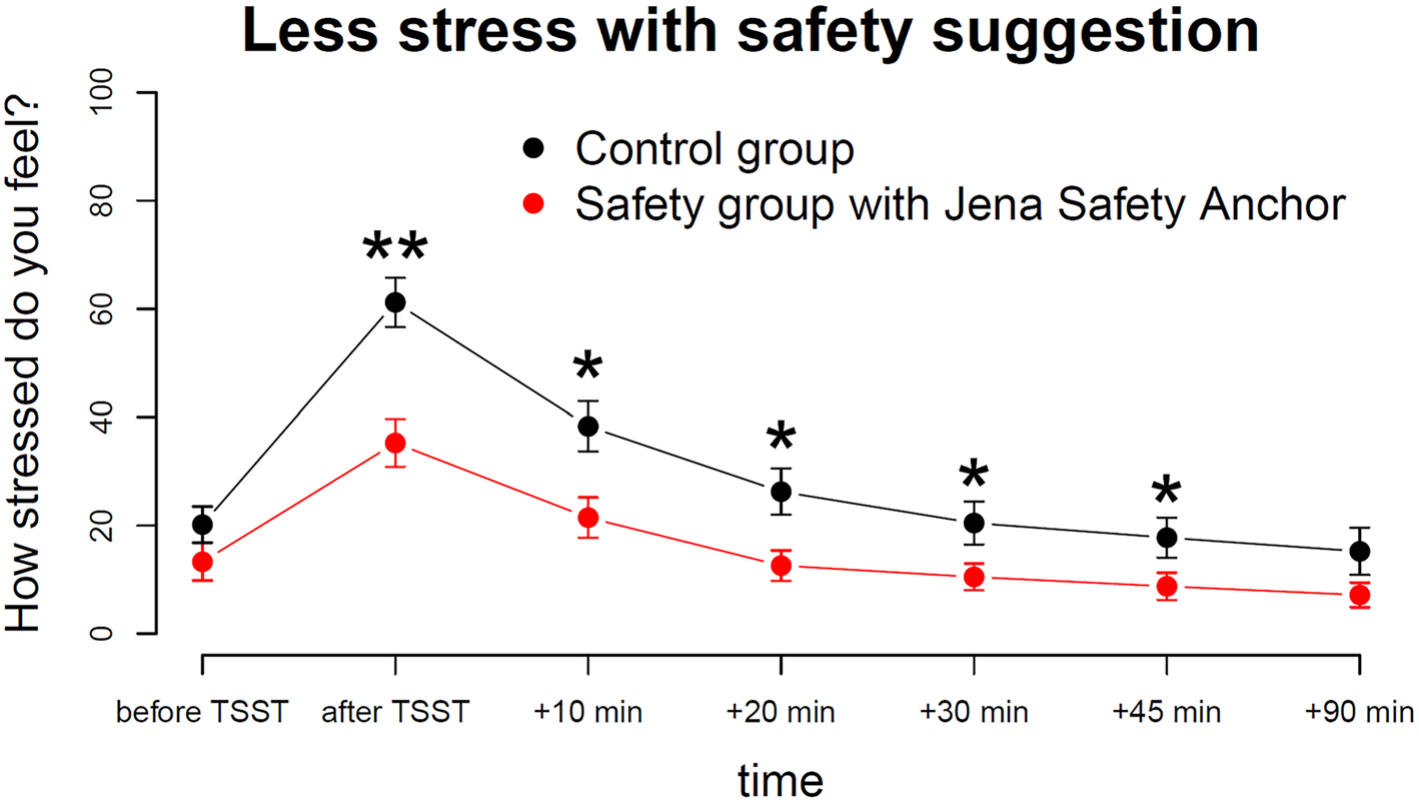
Participants in the safety group reported lower stress levels than participants in the control group, most pronounced immediately after the TSST.

Participants rated their stress levels at seven occasions throughout the main experimental session on a visual analogue scale ranging from 0 (no stress) to 100 (highest possible stress). An ANOVA on the stress ratings with between factors group (safety, control) and suggestibility (low, middle, high) and within factor time (1–7) revealed a significant main effect of group, *F*(1,53) = 8.2, *p* = 0.006, a significant main effect of time, *F*(6,323) = 70.8, *p* < 0.001 and a significant interaction of group and time, *F*(6,323) = 4.5, *p* < 0.001. All other effects did not reach significance (*p* > 0.1). Post-hoc *t*-tests show that groups differed significantly at all times except for baseline, that is, before they were handed their S or X paper, and the last time point 90 min after the TSST. The largest group difference appeared immediately after stress exposure as visible in Fig. 3 with two asterisks. The effect size of stress reduction via the post-hypnotic safety suggestion, measured as the group difference in stress levels immediately after TSST, was Cohen’s *d* = 1.06 indicating a large effect.

**Figure 4:**
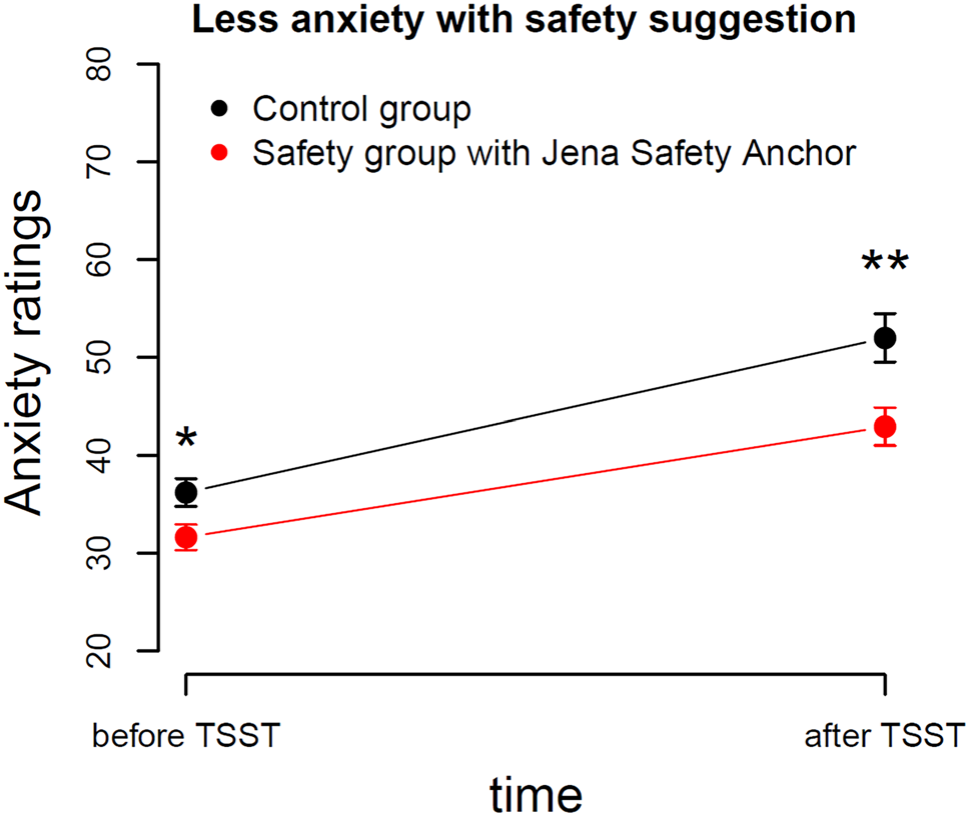
Participants in the safety group reported significantly lower anxiety levels compared to the control group at both time points. The interaction effect of group and time did not reach significance.

An ANOVA on participants’ state anxiety ratings with between factors group (safety, control) and suggestibility (low, middle, high) and within factor time (before TSST, after TSST) revealed a significant main effect of group, *F*(1,52) = 9.1, *p* = 0.004, and a significant main effect of time, *F*(1,52) = 83.5, *p* < 0.001. All other effects did not reach significance (*p* > 0.1). Figure 4 shows that anxiety levels increased after stress exposure, but less so in the safety group. Participants of the two groups already differed in state anxiety before TSST started. Please note that we measured participants’ trait anxiety scores before the main experimental session with the STAI-T, which showed no pre-experimental differences in trait anxiety (see procedure section). Even though trait anxiety levels did not differ between groups, state anxiety levels before the TSST indicate that the safety group felt significantly less anxious compared to the control group before the stress task started. The interaction effect of group and time did not reach significance, so we cannot conclude that the Jena Safety Anchor significantly reduced anxiety.

### Less negative thoughts after TSST and 1 week later

**Figure 5:**
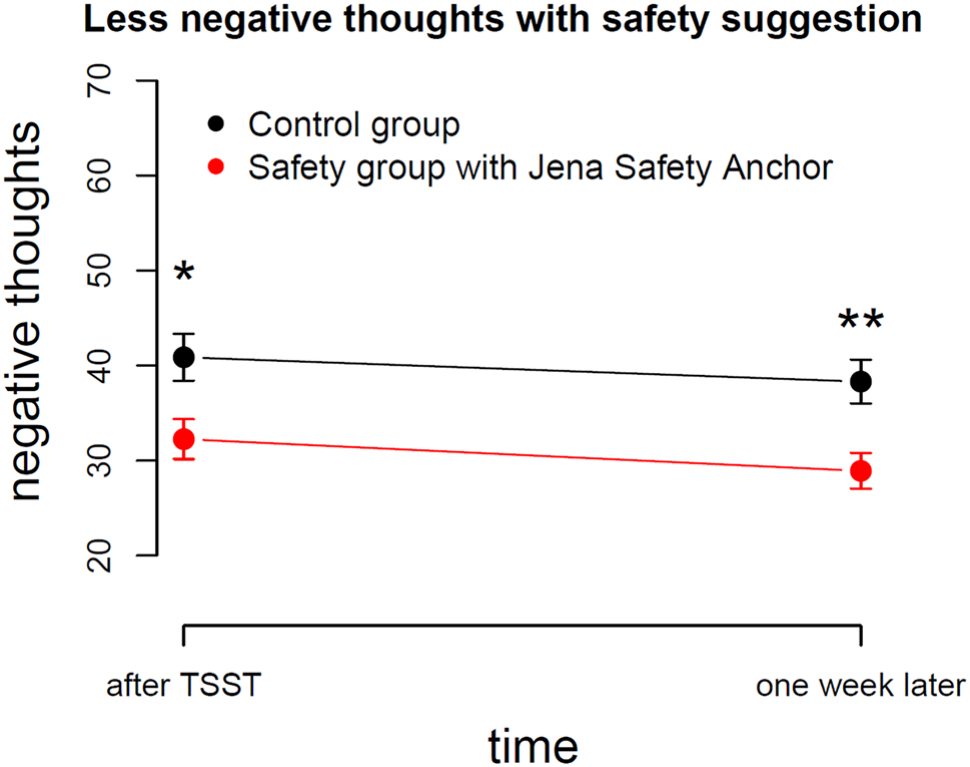
Participants reported fewer negative thoughts in the safety group compared to the control group, 30 min after the TSST and 1 week later.

We asked participants about their positive and negative thoughts 30 min after the TSST and 1 week later in an online survey. Groups did not differ concerning the number of positive thoughts 30 min after the TSST (*p* = 0.8), but the safety group reported significantly less negative thoughts than the control group, *t*(56) = 2.6, *p* = 0.01 with a medium effect size of Cohen’s *d* = 0.7. We observed the same pattern of results 1 week later, with an even larger group difference for negative thoughts, *t*(56) = 3.2, *p* = 0.003 showing a large effect with Cohen’s *d* = 0.8, see Fig. 5.

### No cortisol response to the TSST in both groups

We took six saliva samples over the time-course of the testing session. An ANOVA on cortisol levels with between factors group (safety, control) and sex (female, male) and within factor time (1–6) revealed a significant main effect of time, *F*(5,280) = 8.7, *p* < 0.001. All other effects did not reach significance (*p* > 0.3). We analyzed the difference in cortisol levels at baseline (before the TSST) and 10–20 min after the TSST, typically the time-point of peak stress-induced cortisol release^33^. We found a mean cortisol increase of 0.7 nmol/l from baseline to peak. This is lower that what has previously been defined as a physiologically significant cortisol increase from baseline levels (i.e., 1.5 nmol/l^32^), suggesting that our participants showed no relevant cortisol stress response. Please go to our Zenodo repository (10.5281/zenodo.10561059) to see the figure showing cortisol levels over time for both groups and the raw cortisol data.

### Adrenaline and heart rate increased after TSST in both groups

We took four blood samples over the time-course of the study to measure adrenaline levels. An ANOVA on adrenaline levels with between factors group (safety, control) and sex (female, male) and within factor time (1–4) revealed a significant main effect of sex, *F*(1,56) = 5.4, *p* = 0.03, and a significant main effect of time, *F*(3,168) = 18.7, *p* < 0.001. All other effects did not reach significance (*p* > 0.09). Male participants showed higher adrenaline levels than female participants (mean male: 41.1 pg/ml, mean female: 27.0 pg/ml). After acute stress, adrenaline levels significantly increased in all participants, *t*(59) = 4.3, *p* < 0.001. The mean rise in adrenaline levels from baseline to immediately after stress was 17.2 pg/ml. Please see our Zenodo repository (10.5281/zenodo.10561059) for adrenaline figures and raw data.

An analysis of participants’ heart rate via seven five-minute time bins over the course of the study revealed that heart rate increased especially during the TSST job interview (mean: 104.4 bpm) and math test (mean: 105.3 bpm) and decreased again afterwards. An ANOVA on heart rate with between factor group (safety, control) and time (7 intervals) showed a significant main effect of time, *F*(6,348) = 128.4, *p* < 0.001. All other effects did not reach significance (*p* > 0.9). For figures and raw data, please check our Zenodo repository (10.5281/zenodo.10561059).

### Hypnosis control item, hypnotic depth and longevity of post-hypnotic suggestion

To verify whether participants were hypnotized, we included a hypnosis test item and a questionnaire measuring hypnotic depth. For the hypnosis test item, participants imagined a heavy weight in their hand that pulls their arm down. All but four participants (56 out of 60) passed the hypnosis test item and followed the suggestion to lower their arm. The exact wording of the entire hypnosis session is available online (10.5281/zenodo.10561059). As an indicator of how deep their hypnotic trance was, participants filled in the ISHD^30^. The mean ISHD score was 92.7 (SD = 15.3), indicating a medium to deep trance state according to Riegel et al.^30^. Participants’ suggestibility scores obtained in the HGSHS correlated significantly with ISHD trance depth in the main experimental session, *r* = 0.54, *p* < 0.001.

Participants rated how safe they felt on a scale from 1 (no change) to 5 (very safe) immediately after the hypnosis session, after the TSST and 1 week later. We did not observe significant group or suggestibility differences for safety ratings (*p* > 0.3). After the TSST, all participants felt less safe than after hypnosis, *t*(59) = 9.1, *p* < 0.001. Looking at the absolute values, participants indicated that they felt safe with the Jena Safety Anchor, both immediately after the hypnosis session (mean: 3.9 out of 5) and 1 week later (mean: 3.04 out of 5). The deeper participants’ hypnotic depth measured with the ISHD, the safer participants felt after the hypnosis session, *r* = 0.53, *p* < 0.001.

## Discussion

In this study, we tested whether one hypnosis session is sufficient to establish a long-lasting anchor of a feeling of safety. During hypnosis, participants wrote the letter S for safety on a piece of paper, and the hypnotist suggested that every time they look at this paper, they will feel safe again. After the hypnosis session, the hypnotist took the S paper away and gave it back only to participants in the experimental safety group, so they could use it during an acute stress task. The control group instead received a neutral anchor, a piece of paper with the letter X written on it. Participants in the safety group showed significantly reduced perceived stress and negative thoughts after the TSST and 1 week thereafter compared to participants in the control group. After the main experimental session, all participants went home with their safety-inducing S paper. One week later, all participants indicated that this S paper still elicits a feeling of safety. Compared to the mindfulness/hypnosis audio intervention that participants used daily for 1 week in the study by Slonena & Elkins^9^, our intervention was only provided once and produced large effects in reducing stress that lasted over 1 week. We conclude that one hypnosis session lasting about 30 min was enough to establish a post-hypnotic safety anchor that was still effective 1 week later.

The results of this study support previous research that the Jena Safety Anchor produces robust and long-term subjective feelings of safety^22^. In the present study, we showed that the Jena Safety Anchor is effective in acute social-evaluative stress situations and improves recovery from a stressful experience. We therefore conclude that our hypnotherapeutic technique is a promising intervention to promote mental health and establish resilience.

The large effect size in subjective stress ratings was not mirrored in significant physiological group differences in cortisol, adrenaline and heart rate. Previous literature has often reported such discrepancies between subjective and physiological stress markers^34^. In fact, in the majority of studies, there are no significant correlations between cortisol responses and perceived emotional stress variables (75% according to Campbell and Ehlert^34^). For example, a study using a 3-day mindfulness meditation training observed a decrease of subjective stress and an increase in cortisol responses in a subsequent TSST^5^, which is the opposite of what we would expect when stress is reduced. Possible reasons for this discrepancy reach from methodological issues to interindividual differences in the degree of psychophysiological correspondence. In our study, we relied on a long tradition of TSST studies conducted in our laboratories that usually show cortisol responder rates of over 80% (e.g.^35,36^ were conducted in parallel and at the same institute as the current study). In addition, adrenaline and heart rate levels showed significant increases after stress exposure, indicating that the sympathetic nervous system responded to our stress induction. Therefore, we consider our TSST paradigm as methodologically sound.

The lack of an average cortisol response of > 1.5 nmol/l^32^ from baseline to peak stress levels in all participants is remarkable, and suggests that the 30-min hypnosis session just prior to TSST onset may have relaxed participants sufficiently to not respond to the TSST. The relaxing effect of the hypnosis session might have also masked the effect of the Jena Safety Anchor in the experimental group, such that no group effect on cortisol levels emerged. The benefit of the post-hypnotic safety suggestion mainly showed in significant reductions of subjective stress ratings as well as less negative thoughts. All participants knew if they were in the safety or in the control group, so the subjective ratings might be affected by demand characteristics. The lack of an average cortisol response in all participants instead cannot be explained by demand characteristics. We conclude that the hypnosis session might have reduced cortisol stress responses, while the Jena Safety Anchor significantly reduced subjective stress and negative thoughts.

A very promising effect of our post-hypnotic safety suggestion was the significant reduction in negative thoughts 1 week after participants did the TSST. The fact that even a week after participants used the Jena Safety Anchor, they still report fewer negative thoughts than participants who did not use the Jena Safety Anchor could be a sign of effective emotion regulation that might prevent rumination and depressive thoughts in the aftermath of a stressful experience^37^. One key element of resilience is to bounce back after acute stressful experiences^3^, so fewer negative thoughts may be an indicator of resilience.

### Limitations

As it was not possible to conduct the study as fully blinded, participants knew in which group they were as did the first author of the manuscript who was the hypnotist and main experimenter. That means demand characteristics might have contributed to participants’ responses. But the TSST jury did not know if the participants used the Jena Safety Anchor during acute stress as well as the person taking saliva and blood samples during the recovery period after the TSST. Furthermore, we did not preregister the study officially. As the study is part of a DFG funded project of the first author of the manuscript, the study design was reviewed as part of this funding. As funding was approved, it implies that the study design of the current study was also approved and we conducted the study exactly as proposed in the funding application, which is available via our Zenodo repository (10.5281/zenodo.10561059).

## Conclusion and clinical use

Importantly for clinical use, establishing a post-hypnotic safety anchor does not require an object like a piece of paper with the letter S for safety on it. A simple gesture or keyword is sufficient to elicit a certain feeling, making it available at all times. In our study, we used a piece of paper to control the usage of the post-hypnotic anchor. As all participants went home with their S paper, they were free to use it again, which could also have beneficial impact on the consolidation of the effect. Some participants also reported in the post survey that just remembering the hypnosis session helped them to feel the same feeling of safety and comfort again. This is in line with the general safe-place procedure in hypnosis where positive resources of the patients are re-activated and made available in challenging situations^38^. In our study, we present a standardized 30-min hypnosis protocol that can induce the feeling of safety by activating inner resources like positive memories and make it available during acute stress. The results show large effects on emotional stress responses that endured over 1 week. Therefore, we consider our Jena Safety Anchor a promising tool to improve stress coping and resilience in just one therapeutic session.

## Data Availability

The datasets generated and analyzed during the current study are available in the Zenodo repository 10.5281/zenodo.10561059.
